# Topological Learning for the Classification of Disorder: An Application to the Design of Metasurfaces

**DOI:** 10.1021/acsnano.3c08776

**Published:** 2023-12-18

**Authors:** Tristan Madeleine, Nina Podoliak, Oleksandr Buchnev, Ingrid Membrillo Solis, Tetiana Orlova, Maria van Rossem, Malgosia Kaczmarek, Giampaolo D’Alessandro, Jacek Brodzki

**Affiliations:** † Mathematical Sciences, University of Southampton, Southampton SO17 1BJ, United Kingdom; ‡ Physics and Astronomy, University of Southampton, Southampton SO17 1BJ, United Kingdom; ¶ Optoelectronics Research Centre and Centre for Photonic Metamaterials, University of Southampton, Southampton SO17 1BJ, United Kingdom; § Infochemistry Scientific Center, ITMO University, 9 Lomonosova Street, Saint-Petersburg, 191002, Russia

**Keywords:** metasurface, surface lattice resonance, topological data analysis, plasmonic, disorder, design, optimization

## Abstract

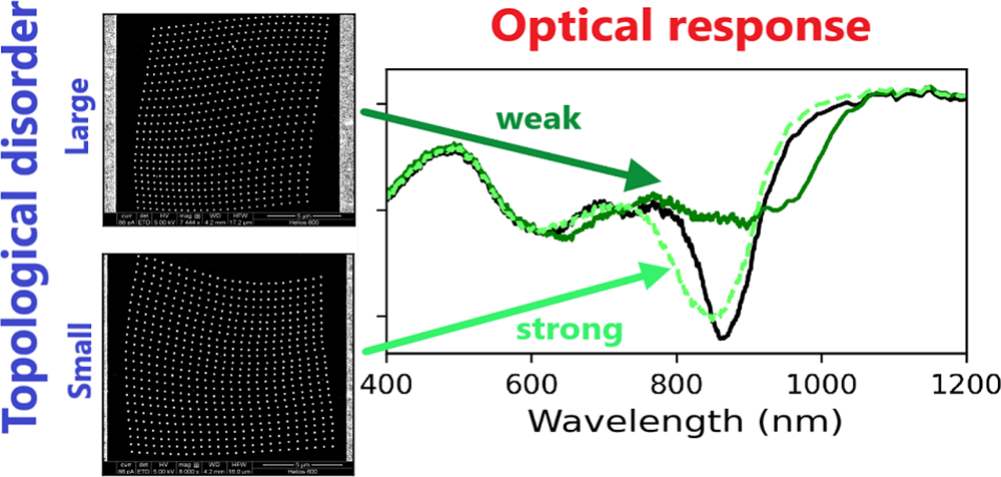

Structural disorder can improve the optical properties of metasurfaces, whether it is emerging from some large-scale fabrication methods or explicitly designed and built lithographically. For example, correlated disorder, induced by a minimum inter-nanostructure distance or by hyperuniformity properties, is particularly beneficial for light extraction. Inspired by topology, we introduce numerical descriptors to provide quantitative measures of disorder with universal properties, suitable to treat both uncorrelated and correlated disorder at all length scales. The accuracy of these topological descriptors is illustrated both theoretically and experimentally by using them to design plasmonic metasurfaces with controlled disorder that we then correlate to the strength of their surface lattice resonances. These descriptors are an example of topological tools that can be used for the fast and accurate design of disordered structures or as aid in improving their fabrication methods.

Metasurfaces are two-dimensional metamaterials with subwavelength scattering elements designed to have electromagnetic properties unobtainable from bulk materials.^1^ However, executing their designs usually requires expensive and time-consuming fabrication methods, such as lithography, limiting their large-scale and large surface area production. In order to circumvent such limitations, significant effort has been devoted to devise quicker and cheaper fabrication techniques, which led to some successes, but usually at the cost of emerging structural disorder. Such examples include gas-phase cluster beam deposition,^2^ nanosphere photolithography,^3^ or lithography-free fabrication methods,^4^ such as bottom-up self-assembled systems,^5−9^ colloid deposition,^10^ or polymer phase separation.^11−13^

While the emergence of structural disorder is usually thought as being an unavoidable downside of these fabrication methods, some photonic-based applications actually benefit from it.^14^ Indeed positional disorder helps to tune^15−17^ or reduce the diffraction,^18^ scattering,^19−21^ reflection,^6,8^ or radiation^17,22^ of metasurfaces, with potential applications in the fabrication of better displays.^23^ Disorder can also suppress grating effects,^24^ make surface-enhanced Raman scattering broadband,^11^ enhance localized photoluminescence,^25^ improve wavefront shaping,^26,27^ and increase light absorption,^28,29^ e.g., for solar cells^12^ or light extraction.^5,30^ For example, coating the air–LED interface with disordered nanostructures provides a broadband coupling between what would have been internally trapped photons to the external radiation, making more energy efficient LEDs.^2^ In some of these systems, correlated disorder is indeed particularly important. For example, a correlation length, induced either by a minimum distance between the nanostructures or by some stealthy hyperuniformity properties, helps to create metasurfaces with larger absorption bands^28^ or broader diffusive properties^19^ or prevent light trapping between nanostructures for more efficient light extraction.^30^

The promising applications of disordered metasurfaces have led to more recent effort to tailor disorder for specific, desired optical properties,^31−34^ for example, using inverse design methods^35,36^ based on machine learning^37,38^ or via topology optimization.^39,40^ Indeed, by combining disorder engineering and topology optimization, one can build metasurfaces with selective light polarization conversion, while minimizing the in-plane phase fluctuation.^41^ Such methods can directly generate optimized disordered patterns, but they can be time-consuming and computationally expensive to implement. In some cases, knowing the link between disorder and the optical properties of a metasurface could significantly speed up the design process by restricting the optimization to the degree of disorder of a metasurface. However, all existing methods to quantify disorder have their strengths and weaknesses, such as being only relevant for specific applications^34^ and being exclusively sensitive to disorder at either long,^42,43^ or short^44,45^ length scales, with some extensions of their scope to medium length scales.^46^ On the other hand, topology has been used to provide insight on physicochemical properties of matter, hinting at potential alternative ways to quantify disorder. Examples of the application of topology include computing graph invariant indices such as the Randić index^47^ and Zagreb indices,^48^ measuring statistics of knots^49,50^ and rings,^51,52^ or using persistent homology.^53,54^ In particular, topological defects are a key element to understand the melting of crystals in 2D into a hexatic phase, by losing translational order, and a fluid phase, by losing orientational order, within KTHNY theory.^55−57^

In this work, we present topology-inspired numerical tools for a comprehensive characterization of disordered metasurfaces. The universality of the tools is established for both correlated and uncorrelated disorder in different systems and structured surfaces. Specifically, they can be used either for the characterization of disordered metasurfaces, built with techniques similar to those mentioned above, or for the fast and accurate design of metasurfaces of specific disorder levels. We applied these tools to design plasmonic metasurfaces, with specific, tailored structural, correlated disorder, originating from randomly generated lattices with different disorder parameters. We illustrate the power of these topological tools by showing theoretically and experimentally that the disorder of the designed metasurfaces, related to the strength of their surface lattice resonances (SLRs), was more accurately represented by our topological measure of disorder than by their generative disorder parameters.

## Results and Discussion

First, a generalized model to generate disorder is presented. We show that a large correlation length may lead to potentially ambiguous designs, where the degree of disorder is poorly represented by the generative/statistical parameters, hinting at the need for better disorder descriptors. Second, the most relevant aspects from topological data analysis (TDA), and the tools required to characterize metasurfaces, are introduced. Using them, it is then shown that disordered metasurfaces with correlated disorder are not well represented by their generative parameters and, instead, are being suitably described by the topological descriptors. Finally, we show the characterization accuracy and predictive properties of these tools by designing metasurfaces with specific disorder levels, first theoretically then experimentally.

### Models of Correlated and Uncorrelated Disorder

A recent work^17^ presented a model of disorder to study how correlated and uncorrelated disorder influence the far-field optical response of a metasurface. We apply our characterization of disorder on their model of disorder that we reintroduce here.

**Figure 1:**
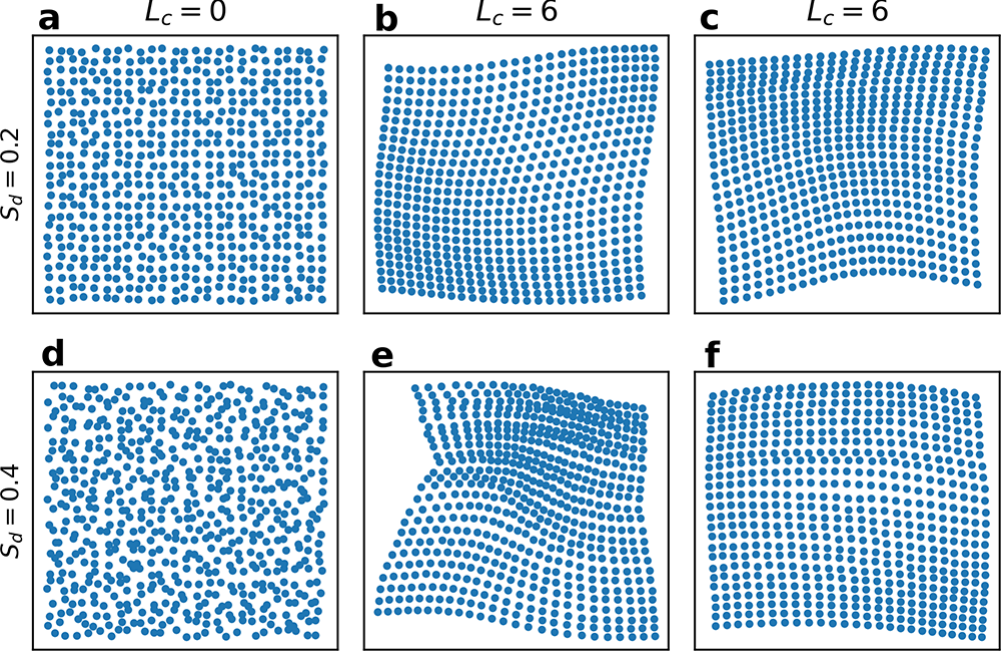
Examples of randomly generated disordered lattices. The top and bottom row lattices are generated using *S_d_* = 0.2 and *S_d_* = 0.4, respectively. The lattices in the left column are uncorrelated, *L_c_* = 0, while those in the middle and right columns have nonzero correlation, *L_c_* = 6.

Starting with a regular lattice, such as a square lattice of period *P* made of *N*_*x*_ × *N*_*y*_ = *N* nanostructures whose positions are defined by , one can define correlated and uncorrelated disorder as follows. Each nanostructure position is modified by a random vector  whose *x* and *y* components are generated from a continuous uniform probability distribution bounded by [−*S*_*d*_*P*, *S*_*d*_*P*]. The nondimensional parameter *S*_*d*_ determines the strength of the uncorrelated disorder. A correlation length can be implemented by adding to  the uncorrelated disorder of nearby nanostructures, indexed by *j*, weighted according to how far they are with a factor *C*_*ij*_,
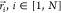
1with *r*_*ij*_ the distance between the nanostructures *i* and *j*, including the uncorrelated disorder applied to  and . The correlation length is given by the full width at half-maximum of *C*_*ij*_, which is equal to , which is proportional to the nondimensional parameter *L*_*c*_. The total disorder perturbation can be summarized as
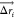
2Using the expression eq 2, we generated lattices with varying values of *L*_*c*_ and *S*_*d*_; see Figure 1. In Figure 1a and d, one can visually appreciate that the strength of uncorrelated disorder, for *L*_*c*_ = 0, is well represented by *S*_*d*_. However, while a nonzero correlation length makes the lattices’ distortion smoother (middle and right columns of Figure 1), it can also make the disorder strength of the lattices ambiguous. For example, one can see that the lattice in Figure 1e is more disordered than the lattice in Figure 1b, as expected from the values of their *S*_*d*_ parameter (0.4 and 0.2, respectively). The same cannot be said about the lattices in the right column. Indeed, the lattice represented in Figure 1c seems more disordered than the lattice represented in Figure 1f, despite being respectively generated with *S*_*d*_ = 0.2 and *S*_*d*_ = 0.4. Therefore, even though the lattices in Figure 1b and c, or in Figure 1e and f, were generated with the same parameters *L*_*c*_ and *S*_*d*_, respectively, the probabilistic descriptor *S*_*d*_ does not provide a clear comparison of their relative disorder strength.

While a nonzero correlation length makes the generative parameter *S*_*d*_ less accurate to represent the positional disorder of a lattice, it also destroys the information about the original regular lattice by inducing collective movements of the lattice’s points. This makes a statistical description of correlated disordered lattices much harder to implement due to not having a reference lattice to compare them to. In order to circumvent such constraints, we introduce topology-inspired numerical tools allowing us to compare lattices with each other in a way that highlights the influence of a correlation length and provides a more accurate measure of disorder than *S*_*d*_.

### Topological Characterization of Disorder

TDA is a collection of tools originating from topology and geometry, designed to provide qualitative and quantitative descriptors of structures in data sets. They have been successfully applied to various systems in different fields ranging from cosmology^58,59^ to solid state physics.^60−64^ Topological measures such as the Randić^47^ and Zagreb indices^48^ rest on the topological properties of graphs, making them particularly interesting when a physical system has natural graph representation, such as molecular compounds. Similarly, statistics of knots provides relevant insight on configurations of elongated objects such as DNA^49^ or proteins.^50^ On the other hand, persistent homology relies on tracking the topological properties of a family of simplicial complexes indexed by a scale parameter, such as a distance or pixel intensity,^65^ thus providing insight on the scale of topological features, with applications for example in material science.^53,54^ We give here a brief description of persistent homology. More detailed, introductory notes can be found in the literature,^66,67^ and the whole process can be executed using standard libraries such as GUDHI^68^ or Ripser.^69^

Starting from a point cloud such as that in panel a of Figure 1, we build a collection of topological spaces called Rips simplicial complexes, indexed by a real number *r*. For a given value of *r*, the complex is constructed as follows. A ball of radius *r* is drawn around each point of the point cloud. If two balls intersect, a link between their respective centers is added. Similarly, higher order links are added to the complex upon the intersection of three or more balls. Restricting ourselves to only two dimensions, which is relevant for flat metasurfaces, circles of radius *r* are drawn around each point and only the connections between pairs and triplets of points are considered. The topological properties of each simplicial complex, the number of connected components, encoded in the homology of degree 0 (*H*_0_), and the number of loops in two dimensions, encoded in the homology of degree 1 (*H*_1_), can be directly computed using algebraic topology. Tracking the evolution of these topological features for different values of *r* provides useful insight into their scale. These features can be summarized in a persistence diagram for which each feature, indexed by the integer *i*, is represented by two coordinates, their “birth”, *b*_*i*_, and their “death”, *d*_*i*_, which are the values of 2*r* at which they appear and disappear.

**Figure 2:**
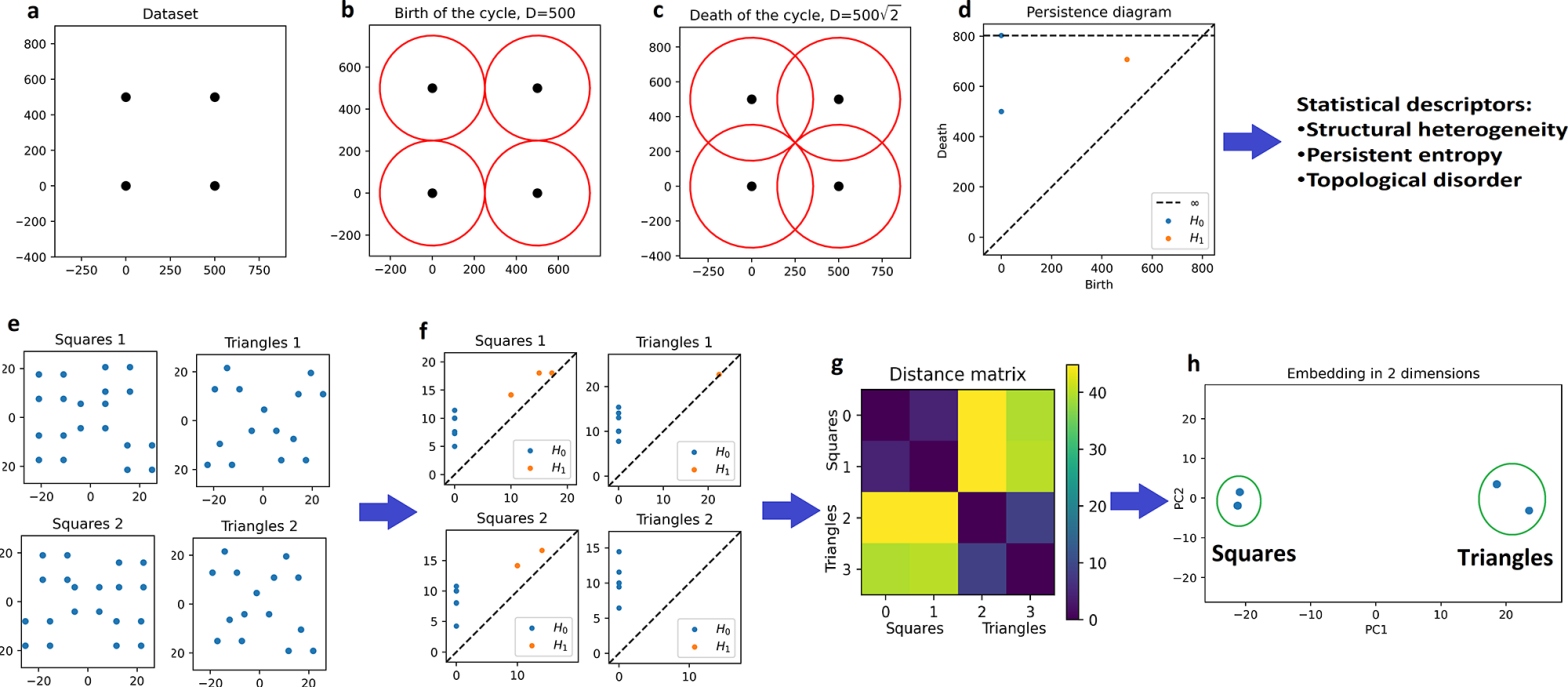
Examples of two key TDA processes used in this paper. The top row represents the computation of persistent homology from the data set (a) to its representation in a persistent diagram (d). In (b) and (c) are represented the circles whose diameters correspond respectively to the birth and death of the loop of this data set (single *H*_1_ point in the persistence diagram in panel d). The bottom row represents the computation of the embedding of data sets (e) in a two-dimensional space (h) via the computation of their persistence diagrams (f) and the distance between them (g). Data sets of the same type are clustered in the embedding space (panel h).

For example, if we consider a simple point cloud such as a square of side 500, Figure 2a, the birth of a loop happens when the diameter of the circles is equal to the side length of the square, Figure 2b. When the diameter of the circles is equal to the diagonal of the square, Figure 2c, the area between the four points is filled. This induces the death of the loop, as it can now be contracted to a single point. The loop is then represented as a point at coordinates (500, 707), labeled *H*_1_, in the persistence diagram, Figure 2d Additionally, four connected components, one for each point of the point cloud, are born at *r* = 0. When the diameter of the circles is equal to the side length, Figure 2b, only one connected component remains as all the points are connected to each other. Therefore, three connected components die when the circles intersect, and they are represented as three points at coordinates (0, 500), labeled *H*_0_, in Figure 2d. The last connected components remain for *r* → *∞*. As the computation of persistent homology was stopped at *r* = 400, we assign to the last connected component the *H*_0_ point at coordinates (0, 800).

This example illustrates how persistent homology can provide interesting insight into KTHNY theory.^55−57^ Indeed, the coordinates of the topological features in the persistence diagrams directly depend on the shape of the points in the point cloud. In particular, if a lattice is made of regular polygons with *N* vertices, each polygon will contribute *N* points labeled *H*_0_ at coordinates (0, *L*), with *L* the distance between the vertices, and 1 point labeled *H*_1_ at coordinates . Topological defects can therefore be effectively tracked in a persistence diagram as they induce a shift of the positions of the topological features.

The second row of Figure 2 illustrates how TDA can be used for the clustering analysis of point clouds. The first step is to measure the “distance” between point clouds, which can be done by measuring the distance between their corresponding persistence diagrams. Several metrics can be defined over the space of the persistence diagrams. We have chosen the Wasserstein distance^70^ for its simplicity of use. If several point clouds are considered, one can build a geometrical embedding, for example, via classical multidimensional scaling,^71^ in which each point cloud can be represented as one point and the distance between each point is given by the distance between their respective persistence diagrams (Figure 2h). This provides a visual representation of the configuration space of the different point clouds and can be used to detect clustering. Classical multidimensional scaling provides an embedding of the set of point clouds in a potentially large vector space. However, by sorting the dimensions by how much the embedding varies in each direction, one can project the embedding in a lower dimensional space while minimizing distortions.

For example, we considered two sets of four point clouds made of either triangles or squares, such as represented in Figure 2e. Upon computing their persistence diagrams (Figure 2f), one can measure the Wasserstein distance between every pair of diagrams. The distances can be summarized in a distance matrix (Figure 2g), where the sets of squares are indexed by 0 and 1 and the two sets of triangles are indexed by 2 and 3. This distance matrix shows that a set of squares seems to be more similar, or closer, to another set of squares than to the sets of triangles, as the distance (0, 1) is smaller than the distances (0, 2) and (0, 3). Similarly, a set of triangles is more similar to another set of triangles than to the sets of squares. This can be directly visualized in their embedding, projected on its two principal components PC1 and PC2, in Figure 2h, where we observe two clusters corresponding to the sets of squares and triangles.

**Figure 3:**
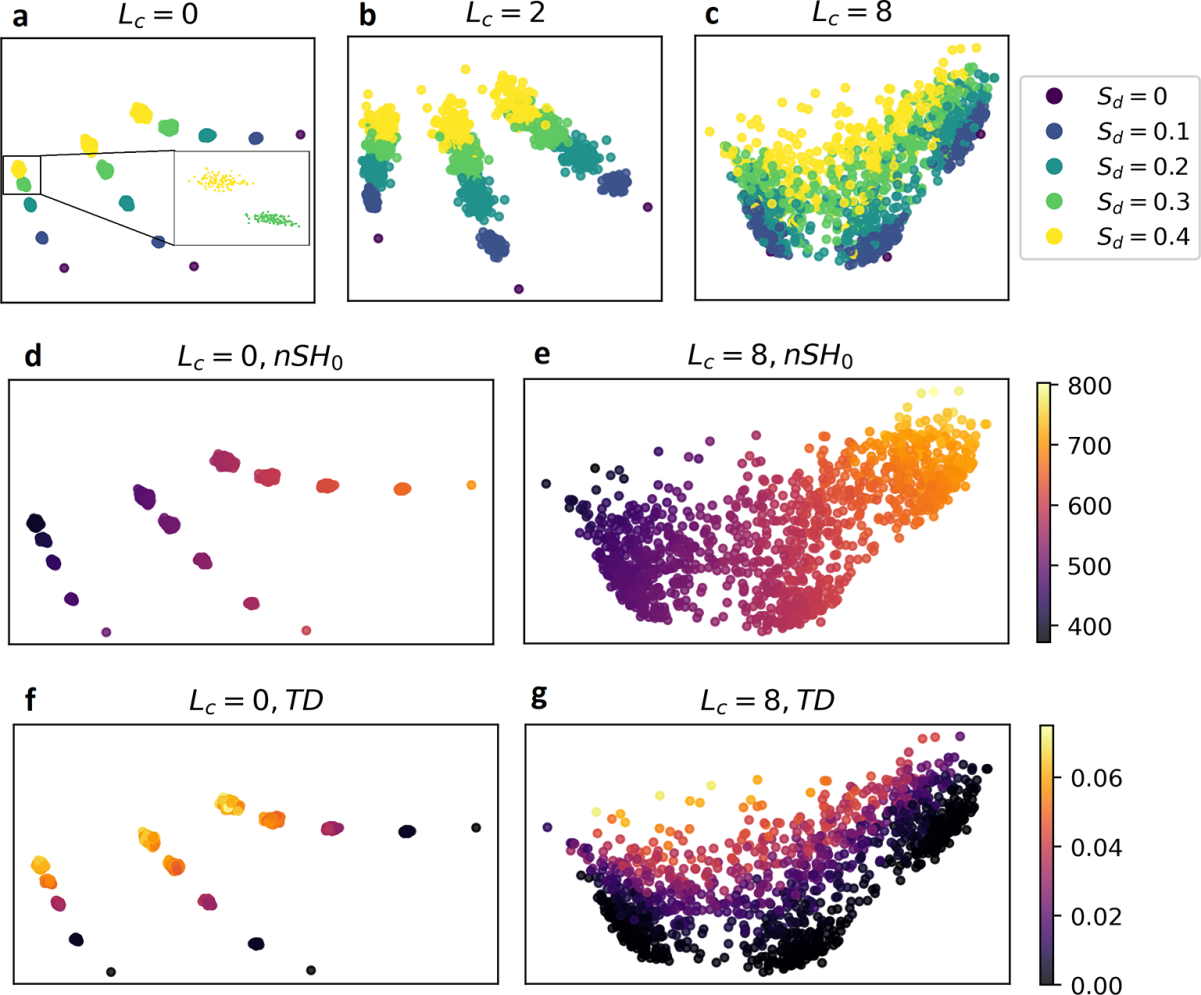
Scatter plots of the two-dimensional embedding of three sets of generated lattices with uncorrelated (a), weakly correlated (b), and strongly correlated (c) disorder. Each set was generated from an original square lattice of period 500, 600, and 700 nm (left to right in panel a) and with *S*_*d*_ ∈ [0, 0.4]. In the absence of correlation, lattices with different values of the period and of *S*_*d*_ are well clustered. In the inset of panel a we adapted the size of points to illustrate how clustered the lattices are. The clustering is lost in the presence of correlations, panels b and c. Panels d and f and panels e and g are equivalent to panels a and c, respectively, with color coding based on the value of n*SH*_0_ (*TD*) in panels d and f (e and g). In both cases the color gradient is not significantly affected by correlation.

In order to visualize the space of configurations obtained from the definition of correlated disordered lattices in eq 2, we performed the embedding of 1203 lattices generated for three different lattice periods, 500, 600, and 700 nm, and five different values of *S*_*d*_ ∈ [0, 0.4]. This was repeated for uncorrelated disorder, *L*_*c*_ = 0, weakly correlated disorder, *L*_*c*_ = 2, and strongly correlated disorder, *L*_*c*_ = 8 (panels a, b, and c of Figure 3). We see in Figure 3a an unambiguous clustering, with a silhouette coefficient of 0.8, of the uncorrelated disorder lattices in terms of their generative parameters, i.e., *S*_*d*_ and the original lattice period. For a fixed value of the period, the lattices appear to live on a simple curve on which five separated clusters of points can be seen, corresponding to the five values of *S*_*d*_ considered. A correlation length increases the size of each cluster, allowing them to overlap, Figure 3b, and therefore reducing the quality of their clustering, with a silhouette coefficient of 0.5. For a large correlation length, like in Figure 3c, each cluster is so large that, effectively, any clustering in terms of their generative parameters is lost (silhouette coefficient equal to 0.001). This is in agreement with what we presented in the previous section, as the configuration space of lattices with nonzero correlation length is much larger, which leads to a far greater variability of the resulting structures despite using the same generative parameters. The expansion of the lattices’ configuration space, proportional to *L*_*c*_, leads to situations where different clusters overlap, like in Figure 3b, which represents lattices generated with different parameters ending up being very similar to each other. Eventually, a large enough *L*_*c*_ makes the overlap between the clusters too significant to accurately represent the lattices as belonging to different clusters labeled by their generative parameters *L*_*c*_ and *S*_*d*_, Figure 3c. Even if one can still see some general trend between the overall lattices’ position and the value of *S*_*d*_ in Figure 3c, one cannot accurately recover the value of *S*_*d*_ of a lattice based on its position. This leads to situations where a lattice generated with a high amount of disorder, i.e., a large value of *S*_*d*_, may be as, or more, ordered than a lattice generated with a small amount of disorder, such as represented in the right column of Figure 1, therefore significantly reducing the accuracy of *S*_*d*_ to quantify the disorder of a lattice.

Using TDA, we were able to overcome the limitation of the parameter *S*_*d*_ to characterize generated lattices. One can build several metrics to describe persistence diagrams, which can be used as simpler descriptors of the topology of data sets or as inputs of more refined machine learning based models.^72,73^ In this work, we use two statistical descriptors based on lattices’ persistence diagrams in order to describe both the typical distance between each point of the lattices and their positional disorder. The first numerical descriptor is normalized structural heterogeneity of degree 0 (n*SH*_0_) and is the sum of the lifetime of the topological features of homology 0, the connected components,^65^ divided by the number of points of the lattice, *N*:
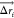
3with *b* and *d* the birth and death of each topological feature of degree 0, *H*_0_, of the persistence diagram . As the death of the topological features of homology 0 is proportional to the distance between the points of the lattices, as seen in the example represented in Figure 2a to 2d, n*SH*_0_ can be directly related to the average nearest neighbor distance between the nanostructures. If one colors the embeddings of uncorrelated and strongly correlated, *L*_*c*_ = 8, lattices of Figure 3 according to the value of n*SH*_0_ of each lattice, we see in Figure 3d that this quantity almost recovers perfectly the periodicity of the lattice for uncorrelated disorder, which confirms our interpretation of the topological features of degree 0. When applied to strongly correlated disordered lattices, Figure 3e, *SH*_0_ provides a smooth ordering of the lattices, following a similar trend to that for uncorrelated disordered lattices.

We also introduce a descriptor called topological disorder (*TD*), inspired from the persistent entropy (*PE*).^74−76^*PE* is defined as
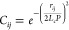
4*PE* is maximal for  and equal to log Ω, with Ω the total number of topological features in . Therefore, *PE* is maximal for regular, periodic lattices and measures how ordered lattices are. In order to avoid the counterintuitive association of a highly ordered lattice with its high persistent entropy, and to define a measure of disorder independent of the lattice’s size, which modifies the number of topological features Ω, we define *TD* as
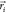
5where the computation is split over the homology degrees *i* in order to capture the fundamental differences between topological features of different homology. Indeed, one can see in Figure 2d that, despite the regularity of the data set in Figure 2a, the topological features in the persistence diagram are located in different places, which would artificially increase the value of *TD*. While the example in Figure 2a is simple, this remains the case for ordered lattices. By construction, *TD* is invariant by rescaling of the typical length of the lattices, making it an orthogonal descriptor of the lattices with respect to n*SH*_0_. *TD* is also minimal for ordered lattices, equal to 0, and is independent of the number of points of the lattices. Therefore, it can be used as a universal measure of disorder, not only for point clouds perturbed from different periodic lattices array but also for point clouds without any inherent order, such as in self-assembled systems. If one colors the embeddings of uncorrelated and strongly correlated lattices of Figure 3 according to their *TD*, we see in Figure 3f that *TD* recovers perfectly the strength of the uncorrelated disorder, regardless of the lattices’ periodicity, which confirms that *TD* is indeed a measure of the lattices’ disorder. When applied to strongly correlated disordered lattices, Figure 3g, *TD* provides another smooth ordering of the lattices, orthogonal to the one given by n*SH_0_*.

These observations suggest that *TD* and n*SH_0_* are two topologically inspired descriptors that can be used to quantify the positional disorder and the typical distance between points of a data set, respectively. Being, by construction, independent of any reference data set, these tools are suitable to classify data sets that are not easily described using classical statistical methods, such as correlated disorder point clouds or self-assembled systems.

### Tailored Metasurface Design, Fabrication, and Spectroscopy

**Figure 4:**
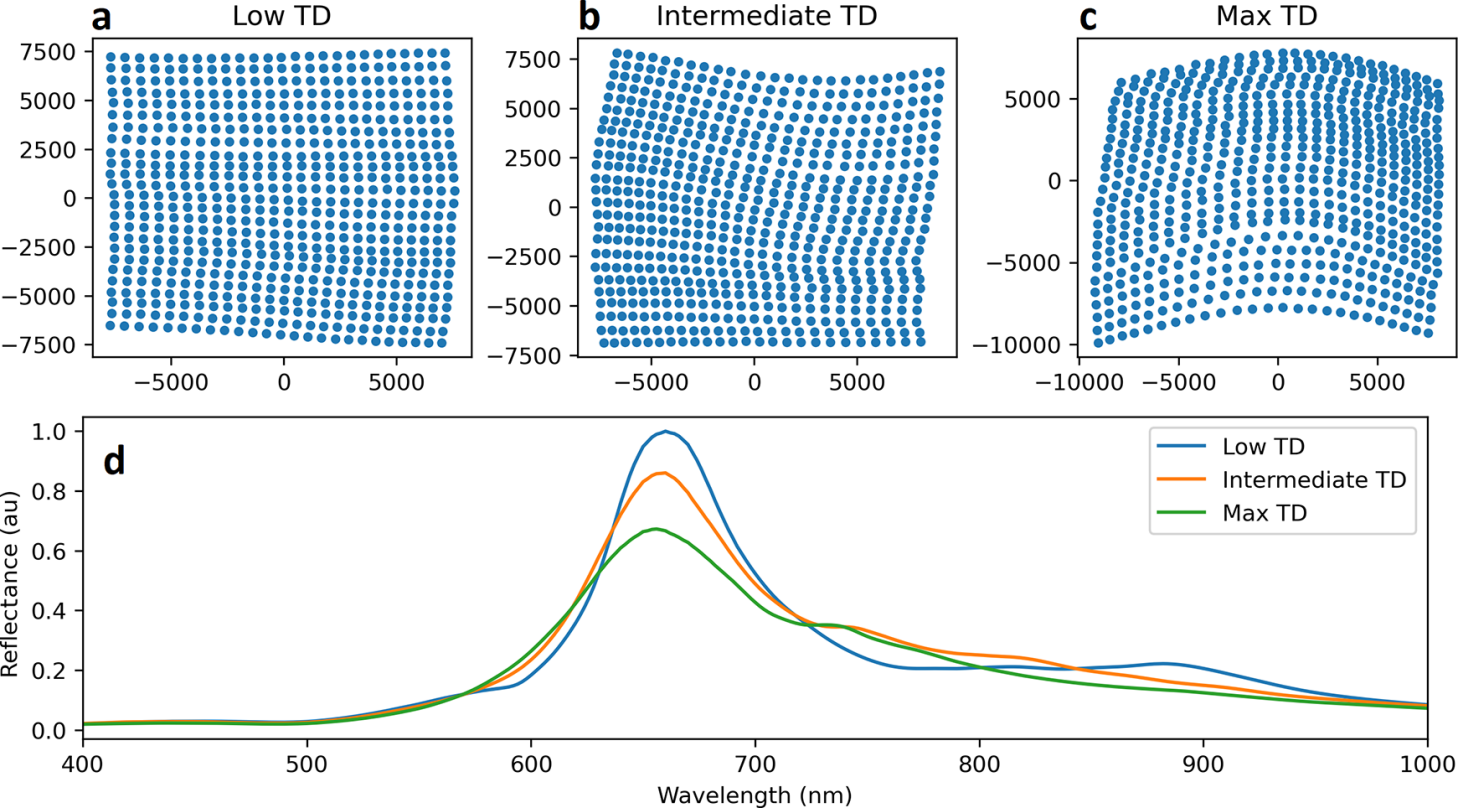
Theoretical investigation of the correlation between *TD* and the strength of SLR. Panels a, b, and c represent respectively the generated metasurfaces of lowest, median, and highest *TD*. Their computed reflectance spectrum, in arbitrary units, is represented in d.

We show the accuracy of *TD* by using it to design, and subsequently build, plasmonic metasurfaces of specific degree of disorder, which we relate to the strength of their SLRs. We first investigate the link between *TD* and the strength of the SLRs theoretically using the discrete dipole approximation.^77^ A total of 1200 lattices of 25 × 25 points were randomly generated with *L*_*c*_ = 8 and *S*_*d*_ = 0.3, starting from a square lattice of period 500 nm, where each point represents the position of a plasmonic nanostructure. The lattices with the highest, lowest, and median value of *TD*, while having similar nearest neighbor distance, estimated by n*SH*_0_, were selected (Figure 4a, b, and c). Each nanostructure is modeled as a gold nanodisk of height 50 nm and diameter 120 nm whose optical properties, under the dipole approximation, are fully determined by their polarizability. The gold nanodisks are assumed to be embedded in a homogeneous glass-like dielectric layer of refractive index 1.41. We numerically compute the reflectance of the three metasurfaces under illumination by a circularly polarized plane at normal incidence, Figure 4d. As predicted, the higher the topological disorder, the weaker the SLRs are. Indeed, one can see on Figure 4d that the amplitude of the SLR dip is inversely proportional to *TD*. Similarly, the quality factors of these resonances are 8.2, 7.5, and 6.5 for the lowest, median, and highest *TD*, respectively.

### Experimental Verification of the *TD*–SLR Link

**Figure 5:**
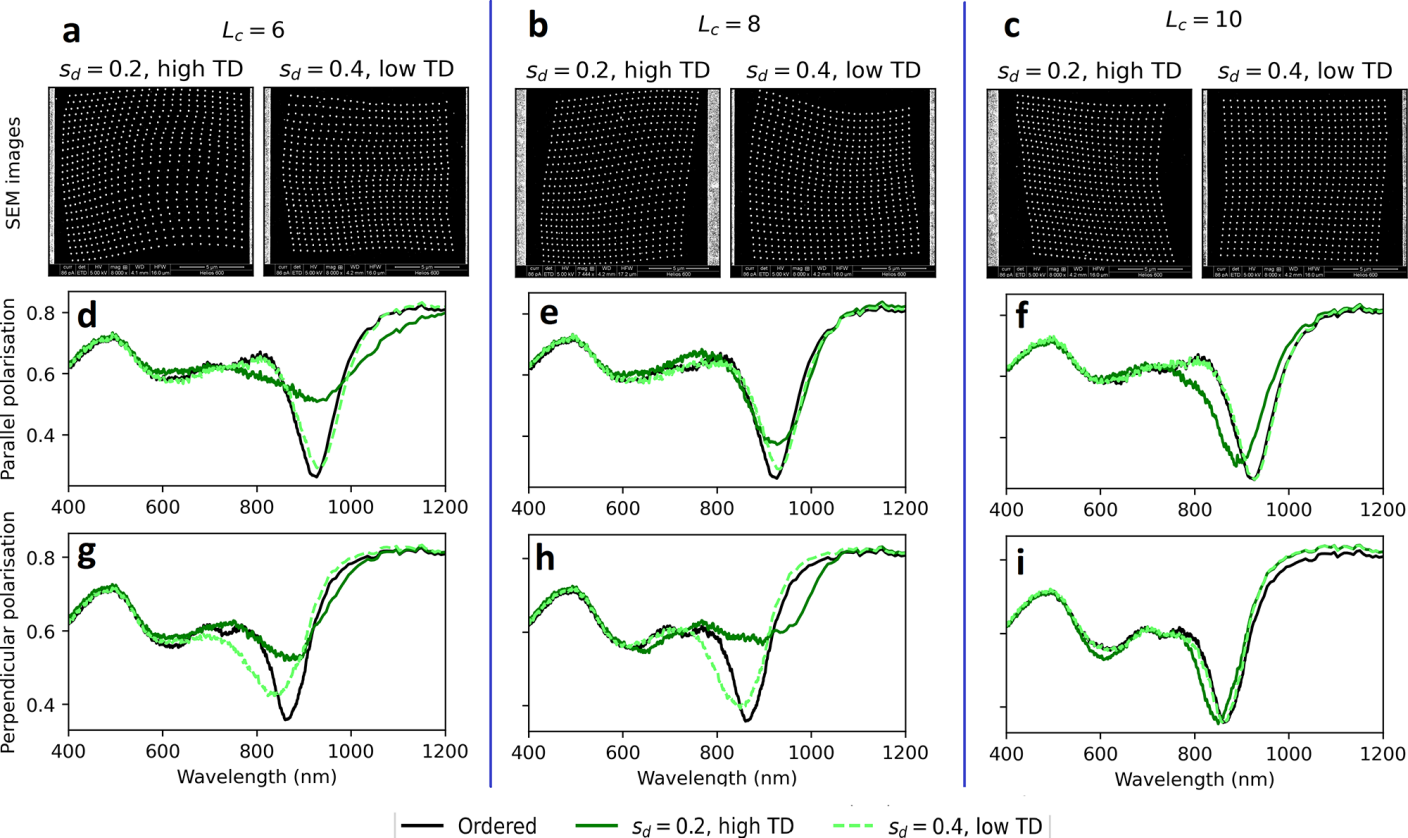
SEM images of the experimental samples (top row) and their transmittance spectra under normal incidence light linearly polarized parallel (middle row) or perpendicular (bottom row) to the long axis of the nanodisks. Each plot displays the spectra of a low and high *TD* metasurface, dashed light green and solid green, respectively, and an ordered metasurface with the same pitch (black). Each column corresponds to the metasurfaces generated with *L*_*c*_ ∈ [6, 8, 10] from left to right.

We additionally experimentally confirmed the link between *TD* and the strength of SLRs by designing metasurfaces built using focused ion beam (FIB) lithography. Using three different correlation lengths *L*_*c*_ ∈ {6, 8, 10} and starting from a regular square lattice of period 500 nm, we generated several hundreds of lattices for two values of *S*_*d*_: 0.2 and 0.4. For each value of *L*_*c*_, two lattices were selected to be compared with each other: the one with the highest value of *TD* among those generated with *S*_*d*_ = 0.2 and the one with the lowest value of *TD* among those generated with *S*_*d*_ = 0.4. Similarly to the previous section, n*SH*_0_ was used to select lattices of similar nearest neighbor distances. We built two sets of seven metasurfaces, three pairs for each value of *L*_*c*_ and one reference square lattice of period 500 nm. The two sets only differ in the size of the nanostructures, which in both cases were elongated 50 nm thick gold nanodisks. The top nanodisk cross-sections are elliptical with *x*- and *y*-axis of size (160, 180) nm and (120, 140) nm for the first and second set, respectively. The resonant wavelength of the SLRs depends both on the distance between the nanodisks and on their polarizability. The latter is strongly affected by the shape of the nanodisks, and their anisotropy induces a shift of the SLRs’ wavelength of up to 60 nm according to the polarization of the exciting light. We therefore report the optical properties of the metasurfaces excited under normal incidence light for two linear polarizations: polarized along the *y*-direction, parallel to the nanodisks’ long axis, and polarized along the *x*-direction, perpendicular to the nanodisks’ short axis. SEM images of the first set, as well as their transmittance spectrum compared to the square lattice, are shown in Figure 5. The results for the second set of metasurfaces, the comparison of these experimental results to the dipolar model, and the SEM images at higher magnification are included in the Supporting Information, in Figures S5 to S8 and in Figure S2, respectively.

The three columns of Figure 5 contain for each *L*_*c*_ the SEM images of the designed pair of metasurfaces (first row) and their transmittance spectra upon excitation by light polarized parallel to the nanodisks’ long axis (second row) and perpendicular to the nanodisks’ short axis (third row). The transmittance spectrum of a periodic metasurface with the same pitch is added for comparison (black lines). We report in Table 1 the quality factors of all the SLRs shown in Figure 5 as well as the *TD* of the corresponding metasurfaces.

**Table 1:** *TD* of the Metasurfaces Reported in Figure 5 and the Corresponding Quality Factors (*Q*) of Their SLRs for Parallel and Perpendicular Polarization of the Exciting Light

lattice parameters	*TD*	*Q* (parallel)	*Q* (perpendicular)
*L*_*c*_ = 0, *S*_*d*_ = 0	0	10.1	11.5
*L*_*c*_ = 6, *S*_*d*_ = 0.2	0.030	4	5.2
*L*_*c*_ = 6, *S*_*d*_ = 0.4	0.012	9.3	6.8
*L*_*c*_ = 8, *S*_*d*_ = 0.2	0.025	6.7	4.4
*L*_*c*_ = 8, *S*_*d*_ = 0.4	0.005	7.8	7
*L*_*c*_ = 10, *S*_*d*_ = 0.2	0.026	8	11.5
*L*_*c*_ = 10, *S*_*d*_ = 0.4	0.002	10.1	11.5

**Figure 6:**
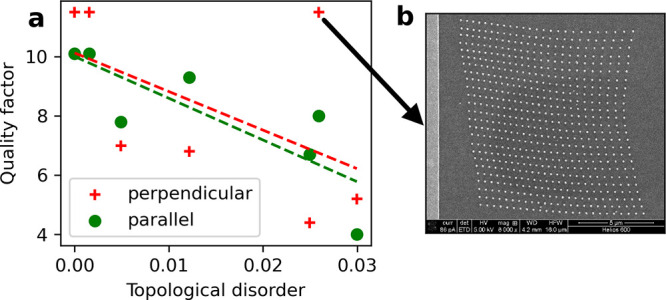
(a) Graph of the quality factors of the SLRs reported in Figure 5 in terms of the *TD* of the metasurfaces under normal incidence light linearly polarized parallel (green dots) and perpendicular (red crosses) to the long axis of the nanodisks. Two best-fit lines are added to represent the general trend of the quality factors for the parallel polarization, in green, and perpendicular polarization, in red, in terms of *TD*. (b) SEM image of the lattice with a high *TD* and a high quality factor, indicated by the arrow.

As can be seen in Figure 5 and Table 1, in five configurations out of six, the SLRs of the metasurfaces designed with a high *S*_*d*_ but a low *TD* are stronger and have a larger quality factor than the metasurfaces designed with a low *S*_*d*_ but a high *TD*, showing that *TD* is indeed an accurate measure of disorder. The only exception is the configuration with *L*_*c*_ = 10 and perpendicular polarization, Figure 5i, for which both metasurfaces have similarly strong SLRs with a quality factor of 11.5, equivalent to the square lattice for this polarization, despite the lattice generated with *S*_*d*_ = 0.2 having a very high *TD* of 0.026. Upon inspecting the lattices of the two metasurfaces generated for *L*_*c*_ = 10, shown in the panel c of Figure 5 or in larger versions in the Supporting Information, one can see that the lattice generated with *S*_*d*_ = 0.4 is ordered, which is reflected in its low *TD* and its high quality factor. However, on the lattice generated with *S*_*d*_ = 0.2 with a high *TD*, also represented in Figure 6b, one can visually appreciate that positional distortion seems to be noticeable only at a large scale, while at short scales, the nanodisks seem to be more regularly spaced as if they were on a square lattice. Indeed, larger values of *L*_*c*_ average out the uncorrelated disorder of neighboring nanodisks, which effectively smooths out the positional shift of each nanodisk, while maintaining large-scale shifts, responsible for the wavy patterns of the two right columns of Figure 1. While this shows that *TD* is sensitive to positional disorder at every scale of the metasurface, the strength of SLR depends mostly on short-scale disorder. Indeed, the interaction strength between the nanodisks decreases as the inverse of the distance between them, under the dipolar approximation, and, effectively, a nanodisk only interacts with a few of its neighbors. Therefore, a metasurface with short-scale order but long-scale disorder, such as the one generated with *L*_*c*_ = 10 and *S*_*d*_ = 0.2 in Figure 6b, can exhibit strong SLRs despite having a large *TD*. This effect can also be visualized if one represents the quality factors of the SLRs in terms of the *TD* of the metasurfaces for both polarizations of the exciting light, Figure 6. We see a decreasing trend of the quality factor in terms of *TD* despite outliers such as the metasurface generated with *L*_*c*_ = 10, *S*_*d*_ = 0.2 that we just commented on and some fluctuations that can be similarly explained as *TD* being affected by large-scale disorder while the quality factor is not. A simple improvement would be to define *TD* locally and also to some defined scale, therefore making it a multiscale measure of disorder, and only consider it up to the scale relevant for optical properties, dependent on the metasurface’s disorder. However, we chose for simplicity to keep the definition of *TD*, eq 5, global in this work.

This illustrates that *TD* is a more accurate measure of the positional disorder of these metasurfaces compared to *S*_*d*_, as in all of the cases reported here, the metasurface that should have been the most ordered, generated with the lowest value of *S*_*d*_, is actually at least as disordered as the metasurface that should have been the most disordered, generated with the highest value of *S*_*d*_. Indeed, while a correlation length, induced by *L*_*c*_ ≠ 0, made *S*_*d*_ more ambiguous to describe the disorder of the metasurfaces, *TD* was able to accurately select lattices of chosen disorder, which we experimentally probed via the quality factor of their SLRs, despite the nonunique relationship between *TD* and SLR quality factors. For comparison, we investigated, in the Supporting Information, the correlation between the built metasurfaces’ quality factors and two standard measures of disorder used in the study of the phase transition of a two-dimensional system, orientational and translational order.^56,57,78^ We found that orientational order provides similar insight to *TD* but overemphasizes the difference between the ordered lattice and the most ordered of the disordered lattices, despite the similarity in their configurations and optical responses. On the other hand, the standard error on the translational order of the disordered lattices is too significant for translational order to accurately quantify their disorder.

## Conclusion

We have shown how topological data analysis and persistent homology can be used to classify both correlated and uncorrelated disordered metasurfaces via their topological disorder. In particular, topological disorder is a significantly more accurate measure of disorder than the generative probabilistic parameters of correlated disorder. We showed, theoretically and experimentally, this accuracy by correlating topological disorder to the strength of surface lattice resonances of metasurfaces made of plasmonic nanostructures, despite the global definition of topological disorder being sensitive to large-scale distortion, while surface lattice resonances are not. While topological disorder can easily be modified to make it a multiscale measure of disorder, the universality, accuracy, and computational speed of its global definition make it an advantageous tool to characterize and tune the fabrication methods of self-assembled disordered metasurfaces, as well as to help design metasurfaces of specific degree of disorder, for example to enhance light extraction for more efficient LEDs or light absorption for improved solar cells. Furthermore, the natural awareness of persistent homology to topological defects suggests interesting future applications of topological data analysis to study phase transitions of two-dimensional systems.

## Experimental/Method

The metasurfaces have a lateral size of approximately 12 × 12 μm and were fabricated in a 50 nm thick film of Au-coated glass substrate using a focused ion beam facility, Helios Nanolab 600 from FEI ThermoFisher Scientific. The metasurfaces were then spin-coated with IC1-200, whose refractive index is similar to that of the glass substrate.

The spectral characterization was performed in transmittance at normal incidence using a microspectrophotometer (CRAIC Technologies) equipped with a tungsten–halogen light source and cooled CCD array.

The persistent homology of all lattices was computed using the Ripser python package.^69^ The computation for each lattice, made of 625 nanodisks, was done in a fraction of a second. The computation of the distance between each lattice’s persistence diagrams considered for Figure 3 was done using the Wasserstein distance from the GUDHI python package.^68^ Embeddings were obtained from the distance matrices by using classical multidimensional scaling. We projected the embeddings in two dimensions for the visual representations in Figure 3. In general such embeddings live in a very high dimensional, non necessarily euclidean, space, and a projection to a two-dimensional flat space can lead to distortions. However, the magnitude of these distortions can be estimated in the classical multidimensional scaling methods by considering the relative absolute value of the eigenvalues of the embedding in each dimension.^79^ For the embedding represented in Figure 3, the eigenvalues of the two largest dimension, used to represent the embedding in 2D, are respectively 278 and 40 times larger than the largest negative eigenvalue, proving that an embedding in a euclidean space is a good approximation. Similarly, the eigenvalues of the two largest dimensions are respectively 22 and 3 times larger than the third largest positive eigenvalue, hinting that a projection in 2D is an accurate visual representation of the embedding.

The numerical simulations of the metasurfaces’ optical properties were done using the discrete dipole approximation^77^ where each nanodisk is modeled as a dipole of the same polarizability. We assumed that the nanodisks were located in a homogeneous dielectric medium of refractive index *n* = 1.41, which is a good approximation of the refractive index of the glass substrate and of the IC1 layer. The reflectance was measured by computing the electromagnetic flux in the direction perpendicular to the surface, assuming a numerical aperture of 0.28, to match the experimental setup. The nanodisks’ polarizability was computed from simulating the optical response of an isolated nanodisk upon excitation by plane waves of different polarizability,^17^ which we performed using the electromagnetic waves, frequency domain interface of the optics module of COMSOL 5.6, solved with a direct solver.^80^

## Supporting Information Available

SEM images of all samples with close-up views; details of numerical simulations corresponding to the fabricated samples; comparison between experimental results and simulations; comparison of the quality factor with the metasurfaces’ orientational and translational order (PDF)

## Notes

A preliminary version of this manuscript have been uploaded on arXiv.^81^
