# Supplementary material for: Topological Learning for the Classification of Disorder: An Application to the Design of Metasurfaces
Source: ACS Nano. 2023 Dec 18;18(1):630–40. doi: 10.1021/acsnano.3c08776 (PMC10796169; doi:10.1021/acsnano.3c08776)
Supplement: Supplementary file 1 — nn3c08776_si_001.pdf [file nn3c08776_si_001.pdf]

Supporting Information for: Topological learning for the classification of disorder: an application to the design of metasurfaces

Tristan Madeleine,[*,†] Nina Podoliak,[‡] Oleksandr Buchnev,[¶] Ingrid Membrillo Solis,[†] Tetiana Orlova,[‡,§] Maria van Rossem,[‡] Malgosia Kaczmarek,[‡] Giampaolo D'Alessandro,[†] and Jacek Brodzki[†]

†*Mathematical Sciences, University of Southampton, Southampton SO17 1BJ, United Kingdom*

‡*Physics and Astronomy, University of Southampton, Southampton SO17 1BJ, United Kingdom*

¶*Optoelectronics Research Centre and Centre for Photonic Metamaterials, University of Southampton, Southampton SO17 1BJ, UK*

§*Infochemistry Scientific Center, ITMO University, 9 Lomonosova street, Saint-Petersburg, 191002, Russia*

E-mail: tm3u18@soton.ac.uk

SEM images

We report in figure S1a an SEM image of one of the fabricated periodic metasurface. In figure S1b and c, we show close up views of the SEM images of four nanodisks, taken from

the two sets of fabricated metasurfaces mentioned in the main document. We show in figure S2 the SEM images of the six disordered metasurfaces of the second set.

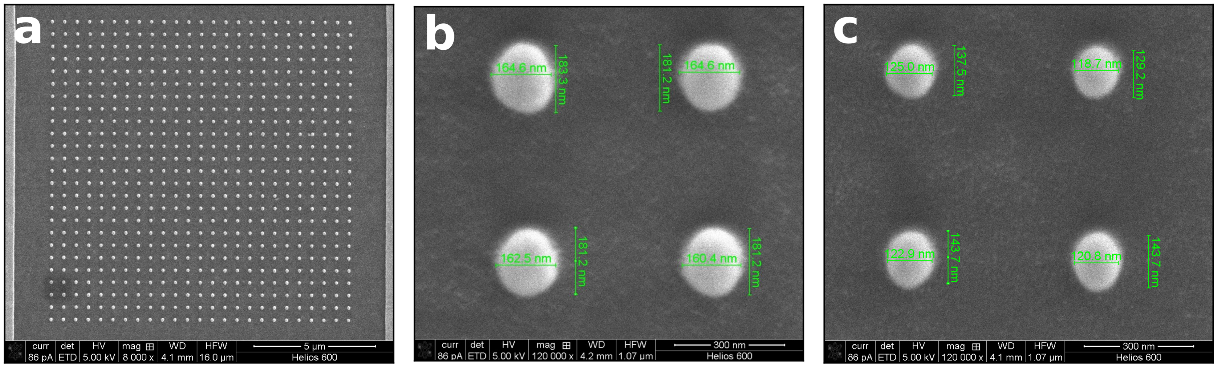

Figure S1: SEM images of a fabricated periodic lattice (panel a). Close up view of the SEM image of the nanodisks built in the first (panel b) and second (panel c) set of samples.

Nanostructure shape

As the images in figure S1 b and c show, the nanodisks have an ellipsoidal cross-section. However, if we assume that the nanodisks have the same ellipsoidal profile across their height, as in figure S3a, we observe a shift of 60 nm of the resonant wavelength between COMSOL simulation and experimental measurements, figure S4. We explain this shift by modelling the nanodisks with a larger base, quantified by the angle between the lateral side of the nanostructure with the normal to the surface, as in figure S3b. We computed the transmittance spectra of periodic array of nanodisks with different angles, figure S4, from which we inferred that the most suitable pillar cross-section shape has an angle of 20 degrees. We repeated this analysis for the two light polarisations, parallel or perpendicular to the long axis of the nanodisks, and for the two sets of samples, and found that an angle of 20 degrees was accurate in every configuration. We therefore assumed a shape of the nanodisks as depicted in figure S3b to compute the polarisability of the nanodisks.

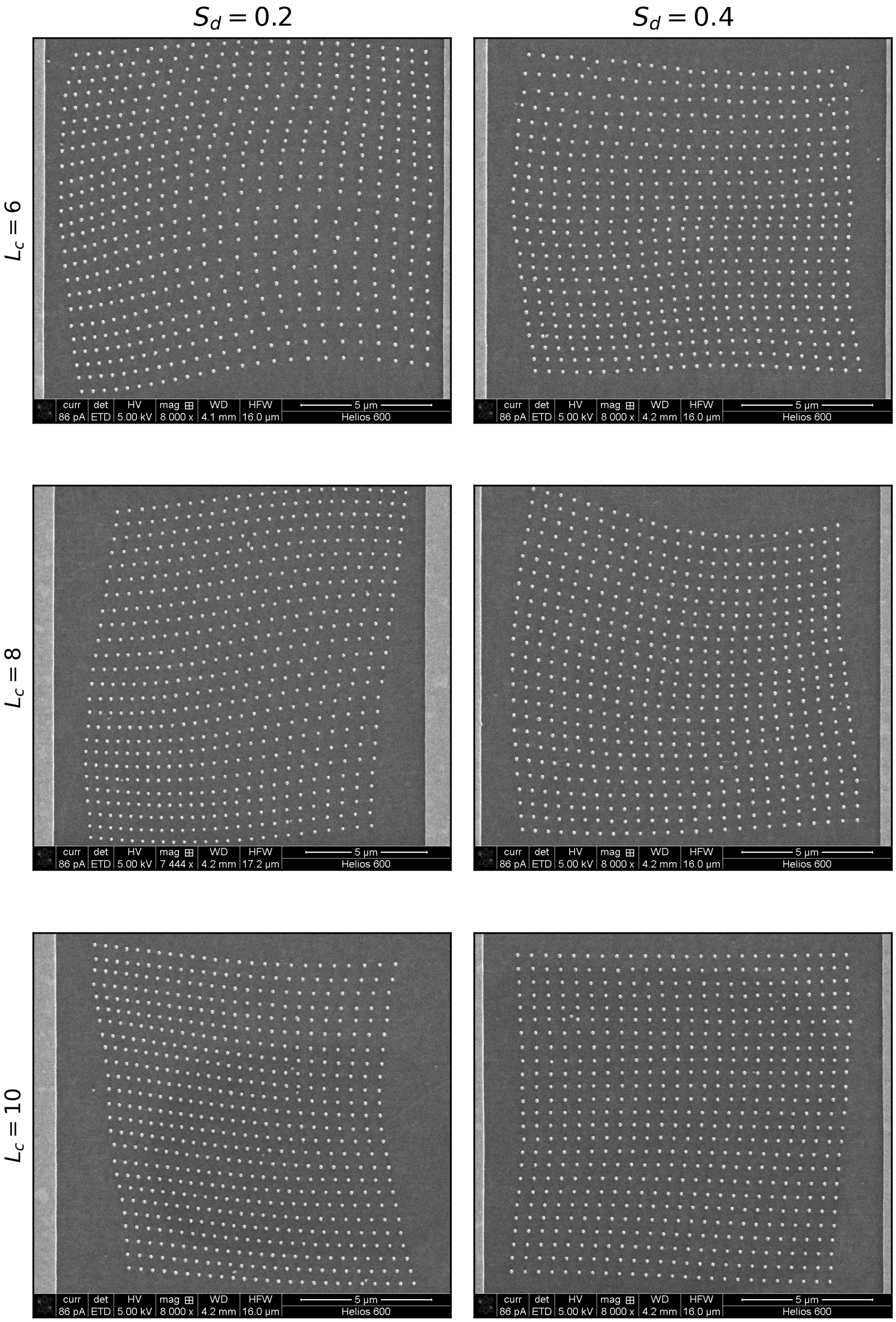

Figure S2: SEM images of the second set of fabricated disordered lattices.

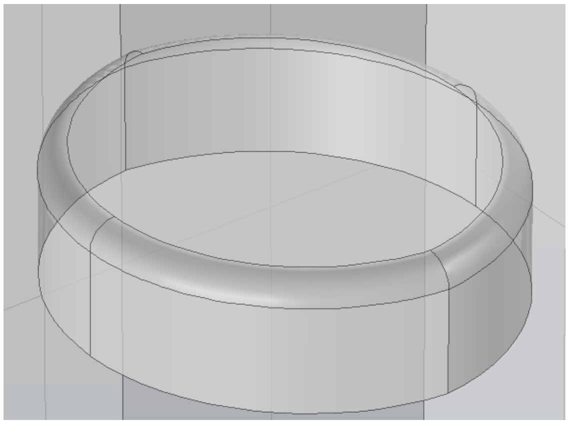

(a) 0 degrees

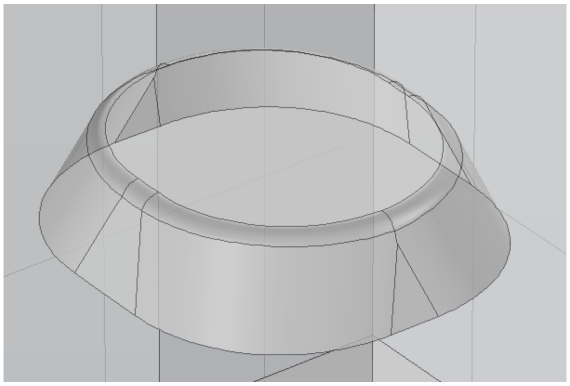

(b) 20 degrees

Figure S3: COMSOL images of the model of the nanodisks for two different angles.

Comparison between numerical simulations and experimental results

From the computed polarisability of the nanostructure, with the shape of the individual nanodisks inferred in the previous section, we simulated the transmittance spectra of the fabricated metasurfaces. We numerically implemented the finite spatial coherence of the exciting light of the experimental setup, about 2 µm, by splitting the metasurfaces into square sub-regions of length 2 µm, following the procedure described in.[1] The transmittance of each sub-regions of the metasurfaces were then added in order to simulate the transmittance spectra of the whole metasurface. We show in figures S5 to S8 the simulated transmittance spectra of the fabricated metasurfaces, compared to their measured transmittance. Figures S5 and S6 correspond to the first set of samples, illuminated respectively with a polarisation parallel and perpendicular to the long axis of the nanodisks. Figures S7 and S8 correspond to the second set of samples, illuminated respectively with a polarisation parallel and perpendicular to the long axis of the nanodisks. Both sets of curves show the same qualitative features, namely the presence of absorption bands in the regular and the low topological disorder lattices. The dipole model is, however, too simple to explore more quantitative agreement. For example, the theoretical dipole surface is, in essence, transparent and does not display

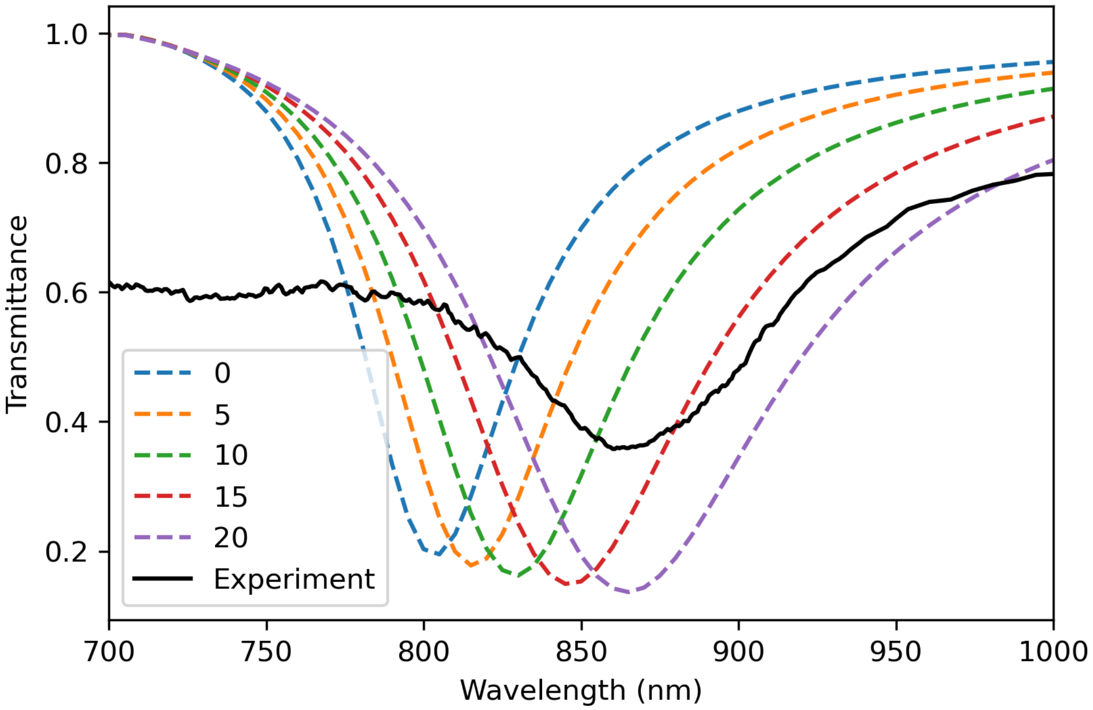

Figure S4: Transmittance spectra of periodic arrays of nanodisks computed for different nanodisks shapes, quantified by the labelled angle, dashed lines. The transmittance spectra measured from the fabricated periodic metasurface is reported as the solid line.

any of the absorption features of the experimental gold disks.

Order parameters

In this section we investigate the accuracy of the orientational and translational order parameters to quantify the disorder of the metasurfaces we built, figure S2. Orientational and translational order parameter are typically used to characterise the phase of matter of two dimensional systems of particles.[2] In particular, the asymptotic behaviour of the correlation of these order parameter can determine if a system is in its crystalline, hexatic or liquid phase. However, due to the limited size of the metasurfaces considered in this work, 625 nanopillars compared to more than 100000 vertices in the literature,[3,4] we chose to only consider the averaged value of both order parameter.[2] The $k-$orientational order parameter

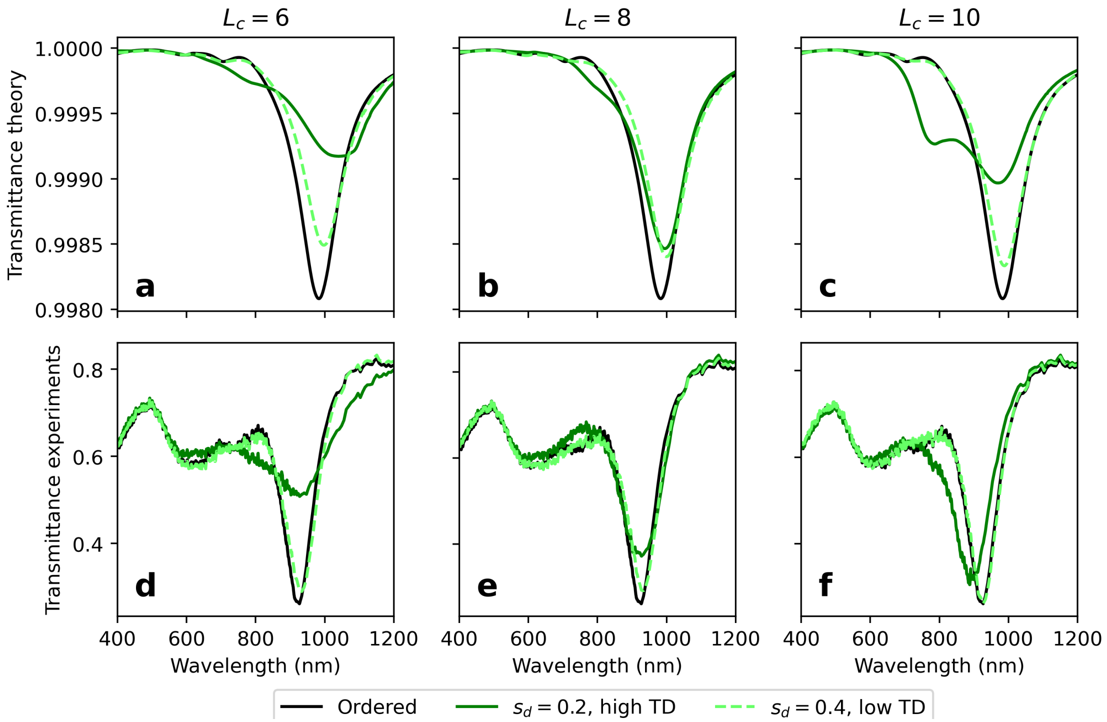

Figure S5: Simulated (top row) and measured (bottom row) transmittance spectra under normal incidence light linearly polarised parallel to the long axis of the nanodisks of the first set of samples. Each plot displays the spectra of a low and high TD metasurface, dashed light green and solid green respectively, and an ordered metasurface with the same pitch (black). Each column corresponds to the metasurfaces generated with $L_c \in [6, 8, 10]$ from left to right.

at the position of the nanodisk i is defined as:

$$\Psi_k(\overrightarrow{r_i}) = \frac{1}{N(i)} \sum_{j=1}^{N(i)} e^{ik\theta_{ij}},$$

where the sum is over the all the $N(i)$ nearest neighbours of the nanostrucure i, found from the Voronoi method.[2] θ_{ij} the angle between the particles i and j with an arbitrary axis, taken to be the $x-$axis in our study. When the system is perfectly ordered with a $k-$fold symmetry, the $k-$orientational order parameter is equal to 1 for each particle of the system. Due to the natural 4-fold symmetry of the lattices we considered, we choose $k = 4$. The orientational order parameter is then defined as the modulus of the average of the 4-orientational order

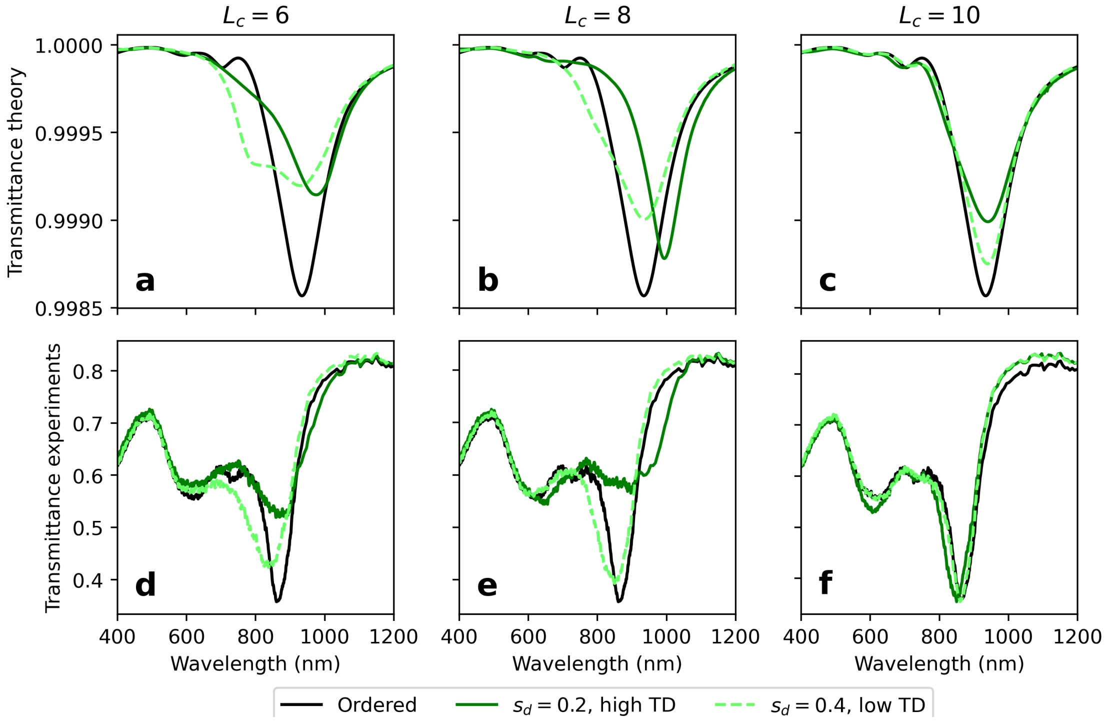

Figure S6: Simulated (top row) and measured (bottom row) transmittance spectra under normal incidence light linearly polarised perpendicular to the long axis of the nanodisks of the first set of samples. Each plot displays the spectra of a low and high TD metasurface, dashed light green and solid green respectively, and an ordered metasurface with the same pitch (black). Each column corresponds to the metasurfaces generated with $L_c \in [6, 8, 10]$ from left to right.

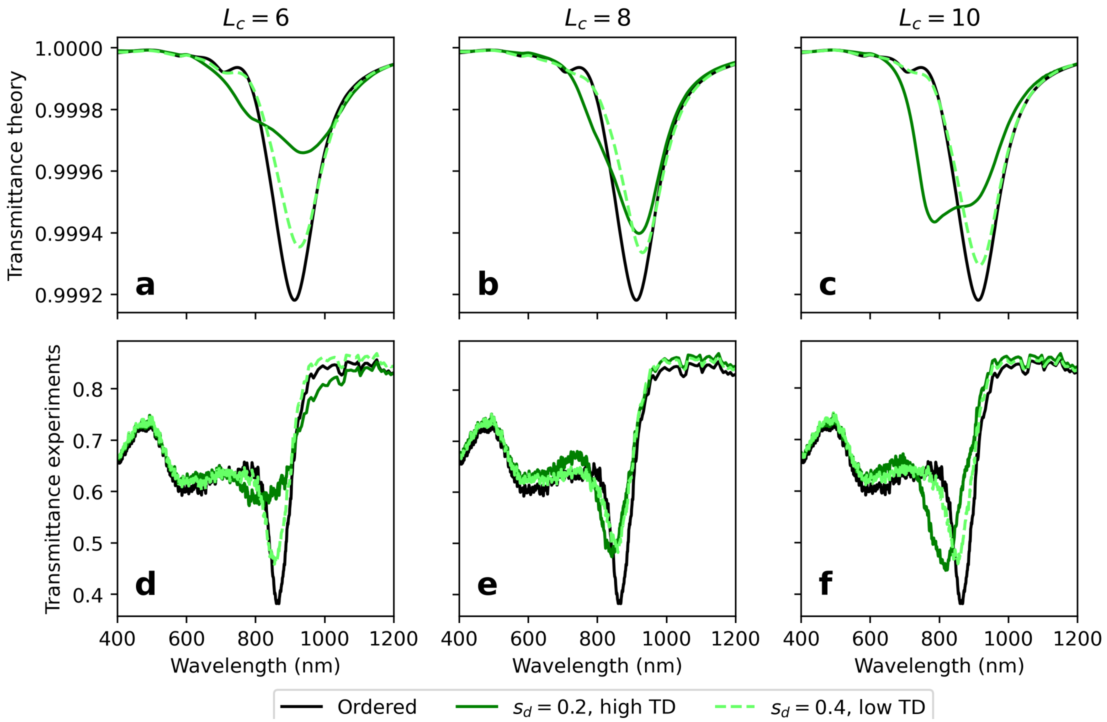

Figure S7: Simulated (top row) and measured (bottom row) transmittance spectra under normal incidence light linearly polarised parallel to the long axis of the nanodisks of the second set of samples. Each plot displays the spectra of a low and high TD metasurface, dashed light green and solid green respectively, and an ordered metasurface with the same pitch (black). Each column corresponds to the metasurfaces generated with $L_c \in [6, 8, 10]$ from left to right.

parameter of all the nanodisks.

The translational order parameter at the position of the nanodisk i is defined as:

$$\chi(\overrightarrow{r_i}) = e^{i\overrightarrow{r_i}\cdot\overrightarrow{Q}},$$

with $\overrightarrow{Q}$ the reciprocal lattice vector of the system, obtained from finding the brightest peak of the structure factor of the lattice.[3] $\chi(\overrightarrow{r_i})$ therefore measures if the particle i fits on an ordered lattice defined by the reciprocal vector $\overrightarrow{Q}$. The (averaged) translational order parameter is taken as the modulus of the average of the translational order parameter of all the nanodisks.

We report the values of the quality factors of the first set of metasurfaces in terms of their

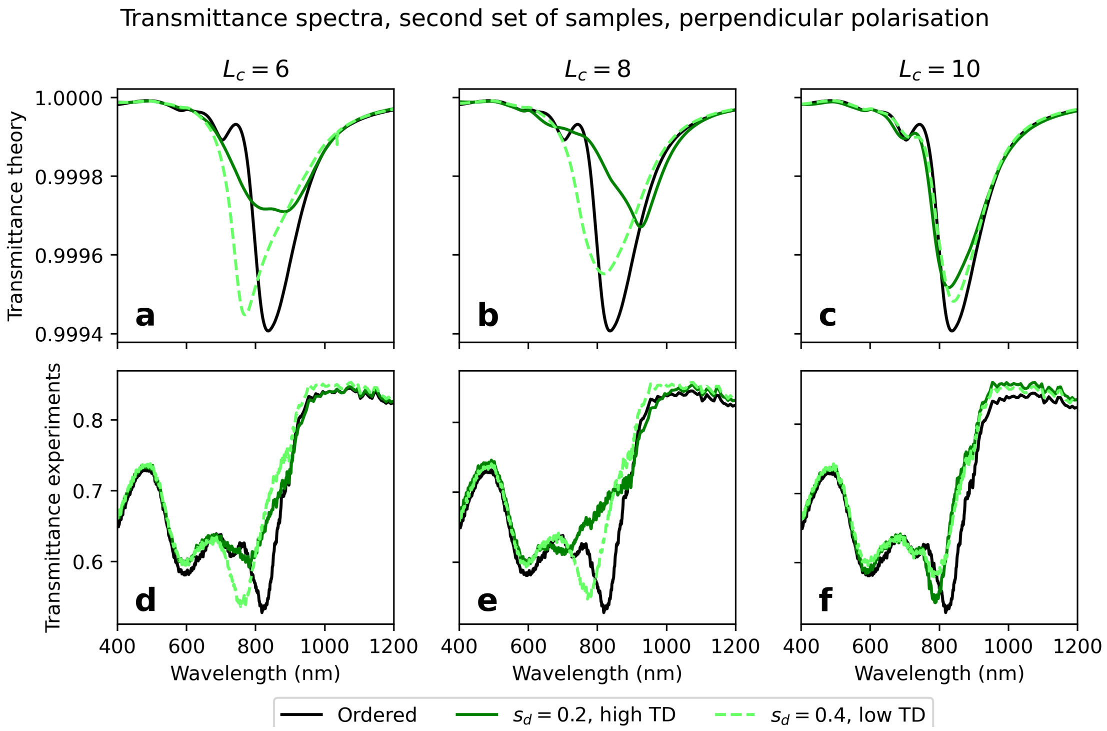

Figure S8: Simulated (top row) and measured (bottom row) transmittance spectra under normal incidence light linearly polarised perpendicular to the long axis of the nanodisks of the second set of samples. Each plot displays the spectra of a low and high TD metasurface, dashed light green and solid green respectively, and an ordered metasurface with the same pitch (black). Each column corresponds to the metasurfaces generated with $L_c \in [6, 8, 10]$ from left to right.

computed orientational, figure S9a, and translational, figure S9b, order parameters, where the standard error of both order parameter is represented by the error bars. We observe an increasing trend of the quality factor of the SLRs in terms of the orientational order, figure S9a, still with the lattice generated with L_c=10 and S_d=0.2 having a relatively small orientational order but a high quality factor, as discussed in the main document.

While this is in agreement with our results on topological disorder in figure 6, the orientational order heightens the difference between the ordered and disordered lattices. For example, all the disordered lattices are clustered in a region of width 0.15 in figure S9a, while the ordered lattice is at distance 0.5 from them. In particular, this seems to overemphasise the difference between the ordered lattice and the most ordered of the disordered lattices,

L_c=10 and S_d=0.4 in figure S2, despite the similarity in their configurations and optical responses. On the other hand, their resemblance is well captured by topological disorder with a difference of 0.002 compared to a maximum difference of 0.03 between the ordered lattice and the most disordered lattice, L_c=10 and S_d=0.4, see table 1.

The standard error of the translational order parameter is too significant for this measure of the order of the lattices to provide any insight. Indeed, while the ordered lattice has a very high translational order, figure S9b, all the disordered lattices have a translational order smaller than 0.1 with error bars so large that one can not faithfully use this measure to determine if one disordered lattice is more ordered than another one.

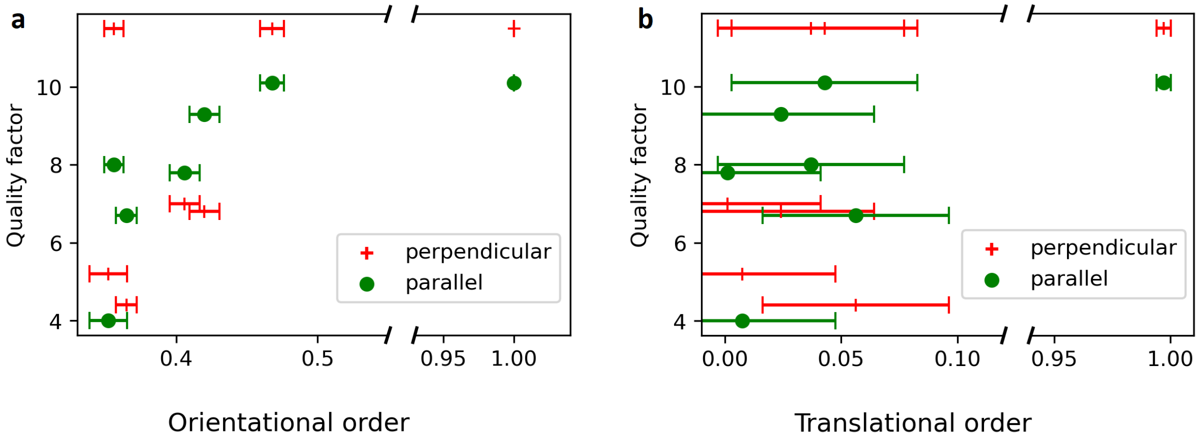

Figure S9: Graph of the quality factors of the SLRs in terms of the orientational order (a) and translational order (b) of the first set of metasurfaces under normal incidence light linearly polarised parallel (green dots) and perpendicular (red crosses) to the long axis of the nanodisks. The error bars represent the standard error of the orientational and translational order.

References

1. Frank, T.; Buchnev, O.; Cookson, T.; Kaczmarek, M.; Lagoudakis, P.; Fedotov, V. A. Discriminating between Coherent and Incoherent Light with Planar Metamaterials. Nano Letters **2019**, 19, 6869–6875, Publisher: American Chemical Society.

2. Dudalov, D. E.; Tsiok, E. N.; Fomin, Y. D.; Ryzhov, V. N. Effect of a potential softness

on the solid-liquid transition in a two-dimensional core-softened potential system. The Journal of Chemical Physics **2014**, 141, 18C522.

3. Anderson, J. A.; Antonaglia, J.; Millan, J. A.; Engel, M.; Glotzer, S. C. Shape and Symmetry Determine Two-Dimensional Melting Transitions of Hard Regular Polygons. Physical Review X **2017**, 7, 021001, Publisher: American Physical Society.

4. Li, Y.-W.; Ciamarra, M. P. Accurate determination of the translational correlation function of two-dimensional solids. Physical Review E **2019**, 100, 062606, Publisher: American Physical Society.
